# Theosophy

**Published:** 1889-01

**Authors:** F. Hartmann


### THEOSOPHY.

BY F. HARTMANN (M. S. T.) IN LE LOTUS.

Translated from the French for HALL'S JOURNAL OF HEALTH by Jules F. Jeaneret.

The word Theosophy is beginning to make a noise in the world, and the public wants to know what Theosophy really is. The answer is probably easier to give than to understand : *Theosophy is knowledge "par excellence."*

To understand this, that which is generally called *knowledge*, and is only imaginary knowledge, must not be mistaken for *real knowledge.* Whatever we know through the teaching of another is only some one else's opinion ; it is not true knowledge. This belongs only to one who knows through himself.

For example every one knows that *one is one.* It is one and not two and the only reason for it is that it is one, neither more or less. It is an evident truth not invented by man ; it is eternal and indubitably imposes itself upon any one gifted with reason. Every one sees it, knows it ; no calculation is needed to demonstrate it. But human reason does not extend much further ; man's intellect lacking the light of intuition does not arrive at the knowledge of the whole truth, and is apt to take refuge in logic and speculation when in need of formulating an opinion upon any hidden truth, and this is called Science. 6 x 6=36 because adding 1 x 1 thirty-six times the same result is obtained as by adding 6x6 six times. If reason was more developed within us, we would know that 6x6 = 36 without need of any calculation, or interrogation about the why of it, simply because our reason would be sufficient to see it and know it. We believe to-day that the teachings we receive at school make learned men of us ; but as I have previously remarked all that which has been conveyed to us through the words of others, constitutes only belief, and very few persons know that 2x2 are 4 through any other means than because they have been told so at school.

When in this wise we examine what true knowledge is, we perceive that all our science, all our philosophy is nothing more than belief.

In this century, believing is not called for ; what is wanted is knowing, yet while rejecting faith, we believe everything without understanding it, provided it is affirmed by a scientific authority, that is to say, by a man thought to be gifted with self knowledge.

We *believe* that the earth is round ; we do not *know* it as long as we are incapable of discovering that fact through the power of our own intellect. We believe it because savants give satisfactory proof of the conclusion that our globe is spherical and not flat as an omelette.

We believe that we know a place called Thibet in the world somewhere, because geography teaches us that such a country exists ; we do not really know it until we have been there, we believe it because we think that were it not a truth it would not be generally accepted as such. There are people who call themselves Theosophists who believe that in Thibet there are *Mahatmas*, they do not know it, but they believe it because Mme. Blavatsky has said so, and they have confidence in the real knowledge of Mme. Blavatsky. Should they suspect that lady of not always telling the truth, the Mahatma's existence would become doubtful to them. Others believe that a Jewess the mother of a man called Jesus, or probably Joshua of Nazareth, remained virgin after having given birth to a son. They do not know it ; they believe it because the Pope has so declared, and they have confidence in the Pope's knowledge. Doubting that knowledge would breed a corresponding doubt about the Immaculate Conception. Should we not believe in the priest's knowledge, we perhaps should doubt the existence of God. Had we no faith in the knowledge of physicians who say it is a good thing to poison the blood through vaccination, we should deny the usefulness of vaccination.

You see therefore that this century is one of belief and superstition. A "Theosophist" who believes in Mahatmas, simply because he has faith in Mme. Blavatsky is no less superstitious than a man who believes in vaccination because he credited the infallibility of a physician.

Science says to us *believe* and gives satisfactory reasons that lead us to believe in that which we can not see. Religion says *believe* ! and gives no reasons for it, pretending that it surpasses our power of reasoning. Theosophy says : *see and know*, open your eyes and try to receive all you can through that light of spiritual intelligence called reason, so that seeing and knowing through yourselves, you will be no more in need of reasoning, of logic or of speculation, nor the authority of fallible and mortal man. Once able to grasp the truth in a direct manner, you will see all about the world which we inhabit ; you will perceive the Mahatmas without leaning upon Mme. Blavatsky's affirmation ; you will understand the mystery of Immaculate Conception, for you will know that the Goddess Nature, is the real mother of the God of the human soul ; that she is an eternal virgin who from the world's beginning has conceived her son by the wholly internal operation of the divine spirit, without any intercourse with an external God.

But to become a true Theosophist, that is to say, to posess that reason which sees and grasps spiritual truth escaping the material vision, what is to be done ? We have already some truths which every one can grasp without effort , these are eternal truths. If instead of always pursuing new illusions, man would pay more attention to these truths and try to understand them intellectually, a great step forward would be made.

One of these truths admitted by every one, but the greatness of which few understand, is *one is one*. It signifies that the universe is only one *omnia in uno* (everything in one) or to borrow the language of the Bible, that there is only one universal God, and that this God is the creating, producing and conserving principal in which we all have our life and being.

There is nothing in the world but God. The ancient Rosicrucians said that all that exists is God, that nothing is in the heavens or upon the earth that is not God. This signified that all the forms and all the forces we know are only various manifestations of a single universal principle under different aspects. In the stones or other primal forms, it manifests no intelligence ; in the animals no conscience, because these forms are not organized for such manifestations. It manifests its wisdom in the human soul, when even man becomes wise.

This universal principle is the *cause* of all that exists and anywhere manifests itself. We call it life, will, ether, space, motion, matter, physical or spiritual light, heat, electricity, magnetism, etc., etc.

All these are God, not Gods, for this word must represent this universal principal only in the sense of its absolute perfection of wisdom, love and intelligence, as when one says, "A divine man," meaning not only a saint, but also a sage, an adept.

If I write this it is not to make you *believe* it, but to help you to find the way through which you will be able to see it and know it by yourselves. What is known or thought to be known, unless acquired through direct knowledge, can only be opinion and nothing more; therefore I beg of you, do not imagine that I can teach you anything you do not already know. I simply tell what my reason has taught me; it is for you to interrogate your own reason and find out the truth or error of what I have said.

My reason tells me—and logic confirms it—that all is *one*. If so, that which is called *matter* and that which is called *force* are one and the same thing under two different states or conditions. We see that comparatively speaking matter is at rest, and that force imparts motion to it, and we can say: Everything that exists is a manifestation of one eternal principle, under two different aspects, one constituting form, the other motion, or everything is produced by the vibrations of the Eternal Principle; when these vibrations are *progressive* they are called *force*, when they are *stable* they are called *matter*.

To satisfy ourselves that this is not a false assertion, examine the relations existing between different forces and different grades of matter, and you will find that by changing the number and direction of these vibrations one force can be transformed into another. You can transform force into matter by changing a progressive vibration into a stable one, and change matter into force by transforming the stable vibration into a progressive one.

For example, if you augment the motion of ether from a thousand vibrations per second to several billions, you will have light instead of sound. You can transform mechanical motion into heat, heat into light, light into electricity, electricity into magnetism, magnetism into chemical affinity, and in the human body, all these forces into yet more exhalted ones. The muscular motion of an early morning walk transforms itself into clearer thoughts; music imparts to the soul emotions which in turn give rise to the corporeal motions observed in dancing, day light gives more gayety than the darkness of night; life and thought are kept up by the food we absorb; thought strengthens the will, the will guides the thought, the thought governs muscular motions, and the will without thought acts upon the instinctive functions of the organism; motions produce emotions, emotions, intelligent activity and vice versa.

Everywhere the corelation of unconscious forces is apparent, and they can be changed one into the other, because these forces are only different vibrations of the one ether which through motion manifests itself into direct forms. Force can be changed into matter, and this transformation occurs at every instant in the human body as well as in the animal and vegetable worlds.

Again, matter can be changed into force under the same conditions. These effects can be experimentally produced. Numerous examples of this are presented to the students of mysticism and alchemy, subjects which our limited space and time do not allow us to consider at present.

Therefore as long as everything is really and finally *one*, it is better and worthier to concentrate our efforts toward knowing that *one* called by us the Eternal Principle, than to simply study its effect upon the visible world. This knowledge of the Eternal Principle is what is called *Theosophy*.

Where can this knowledge be found? The exercise of a power implies its possession. We can not know that which is outside of us. Only that which is in ourselves do we know. This is as true of external objects as of things internal and eternal. If you see a tree, that tree does not enter into our consciousness; we perceive only the image which the rays of light emanating from it project and thus the image enters into our consciousness. If we hear the sound of a bell, the bell does not enter into our consciousness, but the vibrations of the ether give to our consciousness the effect of sound. We imprint a kiss upon one we love; she does not enter into our consciousness, but the well-known sensations attending it make us believe that the loved one is in our arms. The one who really wants to know something, must not lean upon the opinion of another; it is in his own reason that he must look for light. You want to know God! It is useless to seek for him in the churches, but in the interior of your hearts you will find him revealed.

The only temple where God resides, the sanctuary of wisdom, truth love and intelligence, is the soul incarnated in the human body, and we stand nearest heaven whenever we touch the body of either man or woman.

No one can enter into the sanctuary of another one's temple; but every one can penetrate into his own sanctuary. Therefore let us try to enter the sanctuary, so that we shall be initiated, know reason, truth and God, and shall become not only nominally members of the Theosophical Society, but true Theosophists.

It is to be regretted that some of the members of the Theosopical Society, fearing the opinion of the London Society for Psychical Research, have had printed upon the society's program that only a few of the members occupied themselves with the third object, namely the development of psychical powers. We might as well say we are a scientific society, only a few of the members of which occupy themselves with science. There is no other power in man than the divine power, true wisdom. A *Theosophist* who is not a sage or does not desire to become one is an impossibility.

